# Considering the Vulnerable Populations of Young and Old

**DOI:** 10.1177/23800844221141630

**Published:** 2022-12-19

**Authors:** J.S. Feine

**Affiliations:** 1 Oral Health and Society Division, Faculty of Dentistry, McGill University, Montreal, Quebec, Canada

**Keywords:** oral health, caries, early child dental visits, autism, edentulism, pneumonia

It is no surprise that many of the reports in this January 2023 issue focus on 2 of the most vulnerable populations worldwide for poor oral health: children and older adults. Ahmed et al. (2021) describe a quantitative analysis of almost 2.5 million children enrolled in the US Medicaid system. Consistent with previous published literature, their analysis indicates that children who have their first dental visit at the age of 4 y have significantly higher hazard ratios for dental caries (more than 5 times greater) than children who have their first dental visits at the age of 1 y. These findings further support the recommendations of professional groups that children should have their first oral health exam before they reach 12 mo of age.

Regardless, Burgette et al. (2021) used social network analyses, through interviews of mothers of young children, to understand how the information that mothers heard from dentists in their social network agreed with the evidence-based recommendations of professional groups that children should have a first appointment with a dentist by age 1. The authors report that most mothers’ relationships with dentists were in a professional, rather than a social, capacity and that many dentists did not follow these recommendations for children’s first oral health exam, telling the mothers to wait until the age of 3 y. This reluctance of dentists to see children at 1 year of age may be due to a lack of skill or confidence in managing young children. Based on their results, and combined with the results from previous published studies, the authors encourage mandatory infant oral health training in predoctoral dental curricula.

Evidence that many clinicians are reluctant to work with young children at 1 y of age suggests that other populations presenting with behavioral problems might experience the same reluctance.

Parry et al. (2021) carried out a qualitative study in which parents of autistic children and young adults describe the obstacles to dental care that their children have faced and discuss what they think is needed to address these barriers. Based on the parents’ comments, the authors categorized the issues and developed a model of interventions aimed to improve access and care for those with autism. Health services studies in which feedback from the affected population is used as an essential starting point for program development are crucial and more likely to result in a successful program.

Older populations worldwide are rapidly growing. Thus, we need more information and evidence to provide and ensure access to care, as well as appropriate preventive and treatment strategies for this population with diverse needs. Honeywell et al. (2022) measured the number of teeth, as well as the number of pairs of posterior and anterior occluding teeth, and the nutritional status of 305 adults aged 65 to 89 y of age. Consistent with other studies, they found that elders with fewer teeth and occluding quadrants were more at risk of poor nutritional status than those with more teeth and occluding quadrants. Furthermore, in a dose–response fashion, the addition of 1 tooth reduced the odds of malnutrition risk by 3%, and the addition of each occluding pair of teeth reduced these odds by 13%. Having 21 teeth or more in a functional dentition significantly reduced the risk of malnutrition by 88%. This evidence further supports the need to ensure that people retain as many teeth as possible throughout their lives.

While the incidence of complete edentulism is declining, many people are still completely or partially edentulous and wear prosthetic devices to replace the missing teeth. Using an 8-y retrospective longitudinal design, Alzamil et al. (2021) measured the incidence and time to event of pneumonia in a population of independently living older people (65+ y) wearing and not wearing any type of removable dental prostheses (*n* = ~2,300). Among other concerning findings, they report that pneumonia risk was significantly associated with use of dental prostheses and that those who wear dental prostheses have 6 times greater risk of contracting pneumonia than others who do not use dental prostheses. While study limitations somewhat mitigate these findings, they emphasize the need for more research to determine the true risks for pneumonia associated with dental prostheses in older populations.

Although the focus of this editorial was on reports of children’s and elders’ oral health, this issue also offers other interesting articles on a variety of topics, such as chronic pain (Durham et al. 2021), dental implant maintenance in Thailand (Rudeejaraswan et al. 2021), resource allocation for dental services in the United Kingdom (Vernazza et al. 2021), and a meta-regression analysis for heterogeneity in studies on systemic outcomes following periodontal therapy (Oates et al. 2022).

Please enjoy this January 2023 issue of the *JDR CTR*.
